# Clinical and ultrasonographic features of male breast tumors: A retrospective analysis

**DOI:** 10.1371/journal.pone.0194651

**Published:** 2018-03-20

**Authors:** Wei-Hsin Yuan, Anna Fen-Yau Li, Yi-Hong Chou, Hui-Chen Hsu, Ying-Yuan Chen

**Affiliations:** 1 Division of Radiology, Taipei Municipal Gan-Dau Hospital (Managed by Taipei Veterans General Hospital), Taipei, Taiwan, Republic of China; 2 School of Medicine, National Yang Ming University, Taipei, Taiwan, Republic of China; 3 Department of Radiology, Taipei Veterans General Hospital, Taipei, Taiwan, Republic of China; 4 Department of Pathology, Taipei Veterans General Hospital, Taipei, Taiwan, Republic of China; 5 Department of Medical Imaging, Taiwan Adventist Hospital, Taipei, Taiwan, Republic of China; 6 Division of Radiology, National Yang-Ming University Hospital, Ilan City, Taiwan, Republic of China; Technische Universitat Munchen, GERMANY

## Abstract

**Objective:**

The purpose of this study was to determine clinical and ultrasonographic characteristics of male breast tumors.

**Methods:**

The medical records of male patients with breast lesions were retrieved from an electronic medical record database and a pathology database and retrospectively reviewed. A total of 112 men (125 breast masses) with preoperative breast ultrasonography (US) were included (median age, 59.50 years; age range, 15–96 years). Data extracted included patient age, if the lesions were bilateral, palpable, and tender, and the presence of nipple discharge. Breast lesion features on static US images were reviewed by three experienced radiologists without knowledge of physical examination or pathology results, original breast US image interpretations, or surgical outcomes. The US features were documented according to the BI-RADS (Breast Imaging-Reporting and Data System) US lexicons. A forth radiologist compiled the data for analysis.

**Results:**

Of the 125 breast masses, palpable tender lumps and bilateral synchronous masses were more likely to be benign than malignant (both, 100% vs 0%, P < 0.05). Advanced age and bloody discharge from nipples were common in malignant lesions (P <0.05). A mass eccentric to a nipple, irregular shape, the presence of an echogenic halo, predominantly internal vascularity, and rich color flow signal on color Doppler ultrasound were significantly related to malignancy (all, P < 0.05). An echogenic halo and the presence of rich color flow signal were independent predictors of malignancy.

**Conclusion:**

Specific clinical and US characteristics of male breast tumors may help guide treatment, and determine if surgery or conservative treatment is preferable.

## Introduction

The majority of male breast lesions are benign; malignancy of the male breast is rare [1], accounting for only 0.7% to 1% of all breast cancers and 0.17% of all cancers in men [2, 3]. Gynecomastia within the subareolar regions is the most common benign condition of the male breast in newborns, adolescents, and the elderly [2, 3]. Gynecomastia is seen histologically in 50% of all men at autopsy [4]. Other reported benign masses of the male breast include epidermal cysts, lipomas, intraductal papillomas, pseudoangiomatous stromal hyperplasia, granular cell tumors, hemangiomas, schwannomas, myofibroblastomas, and fibromatosis [5, 6].

Men with breast lesions may present with various symptoms and signs that include a palpable breast mass, pain or tenderness, or even nipple discharge. Gynecomastia and the other benign masses usually are palpable and tender [1]. Approximately half of patients with gynecomastia have synchronous bilateral nodules [7]. Men who have one or more of the symptoms and signs often have significant anxiety or emotional distress, as they fear the lesions are malignant [8, 9]. In fact, gynecomastia often resolves spontaneously or upon removal of a causative medication, or treatment of a systemic or endocrine disease [4]. Management of male breast lesions may include fine needle aspiration cytology, excisional biopsy, or even mastectomy, mainly depending on the degree of suspicion of breast cancer, progressive enlargement, persistent gynecomastia, refractory response to medical therapy, and patient requests [4]. However, excessive surgical intervention may result in psychological, physical, and economic burden, and occasionally lead to complications.

Both ultrasound (US) and mammography are significantly more specific than physical examination for the evaluation of male breast masses [7]. Mammography can detect a suspicious mass, suspicious calcifications, and/or architectural distortion of breasts of both sexes, and can evaluate gynecomastia on male breasts [1, 2]. A non-calcified mass is the most common mammographic finding in male breast cancer [2]. However, some case reports have shown that male breast cancers were not detected by mammography [3]. There are no statistically significant differences between the sensitivity and specificity of mammography and US in diagnosing male breast lesions [2, 7]. In addition, a male receiving diagnostic mammography needs to undergo exposure to ionizing radiation, and this is especially inappropriate for adolescents.

Ultrasonography is recommended for the evaluation of male breast lesions because it is a convenient, non-invasive, and low-cost tool that does not require exposure to ionizing radiation [3]. The BI-RADS (Breast Imaging-Reporting and Data System) US lexicon [10] is widely used to describe the characteristics of breast lesions in women. Some US features associated with female breast cancer are irregular shape, nonparallel orientation, non-circumscribed margins, echogenic halo, and increased vascularity in the lesion [11]. However, as breast lesions in men are much less commonly seen than in women, the US features of lesions in the male breast are not well established.

The purpose of this retrospective study was to describe clinical and US features of male breast tumors and their correlations to pathologic findings following biopsies or surgery with a view towards helping to evaluate and manage breast masses in males.

## Materials and methods

### Patients and preoperative clinical presentations

The Institutional Review Board of Taipei Veterans General Hospital (TVGH) approved the study, and waived patient informed consent because of the retrospective nature.

Inclusion criteria for this study were patients with preoperative breast sonographic images and biopsy and/or postoperative pathology of male breast lesions. Once patients received operation in TVGH, surgeon always submit their biopsy specimens or excised tissue to the pathology department in TVGH for examination. Patients with only imaging results or clinical diagnosis were excluded. Biopsy or postoperative pathological examination results served as the final diagnostic standard.

A research assistant blind to the study hypothesis abstracted medical findings and pathology results from the electronic medical record database and pathology database at TVGH using the index terms “male” and “breast”. Over an 8-year period from January 1, 2007 to December 31, 2014, 1,319 male patients had records matching the search terms.

Of the 1,319 patients, 1,110 who did not receive a biopsy or surgery were excluded. Of the remaining 209 patients who received either a biopsy or surgery, 97 were excluded: 11 with inadequate specimens, 19 who were actually females, and 67 without preoperative breast US. Thus, a total of 112 males who had breast US and a postoperative pathological diagnosis were included in the study.

The median age of the 112 patients 59.50 ± 20.19 years (mean age 60.36 years, range 15–96 years), and age was not normally distributed. Preoperative findings included a non-tender palpable lump in 61 (55%) patients, bilateral nontender lumps (one in each breast) in 8 (7%), two lumps in the left breast and one in the right in 1 (1%), bilateral tender palpable lumps (one in each breast) in 1 (1%), areolar swelling bilaterally in 1 (1%), bilateral breast tenderness in 1 (1%), one tender palpable lump in 24 (21%), nontender unilateral areolar swelling in 2 (2%), tender unilateral areolar swelling in 5 (5%), unilateral bloody nipple discharge in 2 (2%), unilateral serous nipple discharge in 1 (1%), a tender lump and serous nipple discharge in 1 (1%), and unilateral breast tenderness in 4 (4%).

### Sonographic examination

Ultrasound was performed from 1 day to 1 week of the physical examination. Technicians with at least 10 years’ experience at the departments of radiology performed the bilateral breast US examinations. Examinations were performed with the patient in the supine position with a scanner using high-resolution linear probes (L12–5 MHz) (S2000 Siemens Healthcare, Mountain View, CA; HDI 5000 and IU 22; Philips Healthcare, Bothell, WA; and LOGIQ E9; GE Healthcare, Milwaukee, WI). The technicians also performed color Doppler US (CDU) if a breast mass was detected, using an appropriate Doppler setting after the gray scale US study. The Doppler gain setting was adjusted to a level associated with minimal noise. The technicians examined a breast in four planes (sagittal, transverse, radial, and orthogonal), and obtained images at the 12 o’clock, 2 o’clock, 4 o’clock, 6 o’clock, 8 o’clock, and 10 o’clock positions, and of the nipple and axilla. If something suspicious was detected, multiple images in different planes were obtained on gray-scale US and CDU. The size, site, and distant to the nipple of lesions were recorded. After the bilateral examination was completed, all images were uploaded to a picture archiving and communication system (PACS) for storage. Revision of static US images was not allowed.

### Sonography interpretation

**Fig 1:**
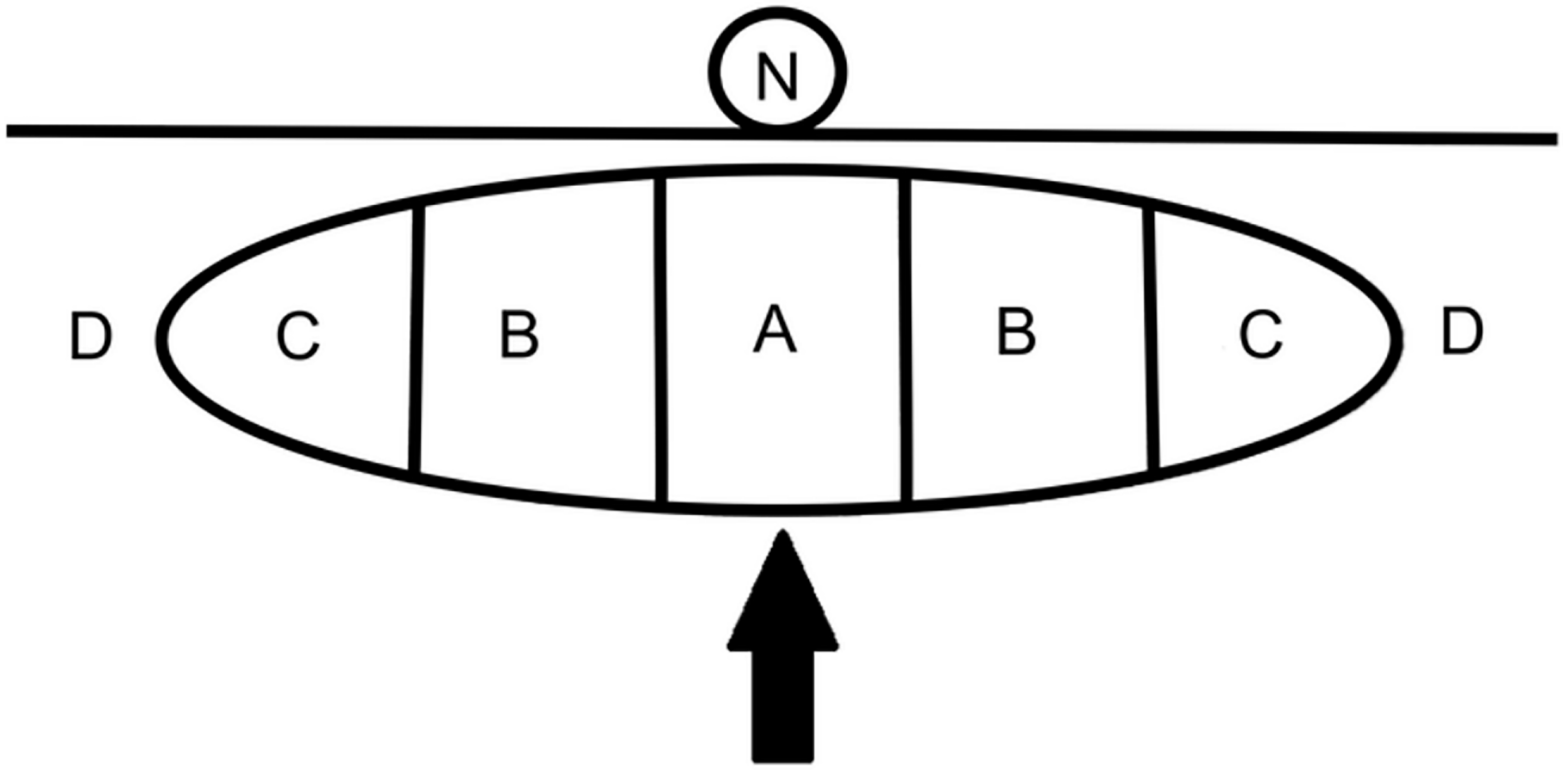
The relationship of a mass to a nipple. The mass (arrow) is divided into five parts of equal width. When part A is located below the center of a nipple, it is classified as concentric to the nipple, as in this example. When part B is below the nipple, it is classified as mildly eccentric to the nipple. If part C is below the nipple or the mass is in area D and not below the nipple at all, it is classified as markedly eccentric to the nipple.

Two experienced radiologists (H-CH, with 20 years of experience, and Y-YC with 10 years of experience), without knowledge of the physical examination findings, the previous preoperative breast image interpretation, or the histopathological results, reinterpreted the preoperative static breast US images of all 125 masses. Final results were determined by consensus. Disagreement were resolved by a third radiologist (Y-HC with 35 years of experience). The following 13 features were recorded for each of the images: maximum diameter of the lesion, site, relationship to the nipple, echotexture, echo pattern, homogeneity, shape, margin, boundary, orientation, posterior acoustic features on gray-scale US, and vascularity and grading on CDU. The lesion diameter (mm) was determined by the maximum diameter shown on US. The lesion site was the right or left breast. For the relationship to the nipple, we divided a mass into five parts of equal width (Fig 1). When the center of the mass (part A) was located under the center of a nipple, the mass was classified as concentric to the nipple. If it was somewhat off center, with part B under the nipple, it was classified as mildly eccentric to the nipple. If either part C or none of the mass was under a nipple, it was classified as markedly eccentric to the nipple.

The echotexture of each mass was characterized as cystic (cystic changes > 75% of a mass), solid (cystic changes < 25% of a mass), or solid and cyst (cystic changes 25–75% of a mass). Color flow distribution was defined as absent, vessels predominantly in the rim, or predominantly internal vascularity. Color flow grading of a mass on CDU was classified as low color flow signal or rich color flow signal. Low color flow signal was defined as the absence of color signals, the presence of occasional transient color pixels, or the presence of only a single color flow signal in or around the mass. Rich color flow signal was defined as multiple color pixels, multiple pedicles of blood supply, or the presence of multiple well-defined vessels in or around the mass. The image with the maximum color flow signals for each mass was chosen for grading.

Other US findings, including echo pattern, heterogeneity, shape, margin, boundary, orientation, and posterior acoustic features, were recorded according to BI-RADS sonographic lexicon classification. The echo pattern relative to fat was classified as anechoic, hypoechoic, isoechoic, hyperechoic, or complex. Heterogeneity was reported as predominantly homogeneous or heterogeneous. Shape was classified as oval, round, or irregular. The margin was defined as circumscribed or noncircumscribed. A circumscribed margin indicated a well-defined or sharp border with an abrupt transition between the lesion and surrounding tissue. The margin of a breast mass was classified as noncircumscribed if the margin had one or more the following features: an indistinct, angular, microlobulated, or spiculated margin. The mass boundary was defined as an abrupt interface or echogenic halo. Orientation was classified as parallel or nonparallel. Parallel orientation indicated that the long axis of the mass was parallel to the surface of the skin or the anterior-posterior diameter of the mass was shorter than the transverse diameter. Nonparallel orientation indicated that the long axis of the mass was perpendicular to the surface of skin or the anterior-posterior diameter of the mass was equal to or larger than the transverse diameter. The posterior acoustic appearance was categorized as normal, enhancement, or shadowing. Because microcalcifications on conventional US are not easily determined during the examination [12], microcalcifications were not recorded on the re-evaluation of the images.

### Histopathologic evaluation

Within 1 day to 5 months of the US examination, all 112 patients underwent a needle biopsy, excisional biopsy, or mastectomy, yielding a total 125 specimens for histopathological examination. This included tissue from one lesion in 100 patients, two lesions in 11 patients, and three lesions in 1 patient. One experienced pathologist (A-FL) with 25 years of experience and without knowledge of the preoperative US findings, retrospectively reviewed all the microscope slides from the 112 patients and confirmed the histopathological results for all 125 lesions.

A fourth radiologist (W-HY) integrated the clinical findings, histopathological results, and the US interpretations of all 125 masses to analyze the clinical and US characteristics, and their association with pathological results.

### Statistical analysis

Data were analyzed with SPSS version 19.0 software (SPSS Inc., Chicago, IL). The level of significance was set at P *<* 0.05. An unpaired t test was used to compare continuous variables (age and maximum diameter), and a χ^2^ or Fisher exact test for categorical variables. Statistically significant US variables were further assessed by multivariate logistic regression analysis, with calculation of odds ratios (ORs) and 95% confidence intervals (CIs).

## Results

### Preoperative clinical findings

Table 1 summarizes the preoperative clinical findings of the 125 breast lesions in 112 male patients. The mean age of the 87 patients with breast benign masses was 56.68 ± 20.51 years (range 15–88 years), and that of 25 patients with breast malignancies was 73.16 ± 12.63 years (range 50–96 years) (P < 0.001). In addition to advanced age, bloody discharge from the nipple was also significantly associated with malignancy (P = 0.0387, Fisher’s exact test). In contrast, bilateral synchronous masses and palpable tender lumps were more likely to be benign (both, 100% vs 0%, P = 0.0037 and P = 0.0019, respectively, Fisher’s exact test). Of the 125 masses, 50 (40%) that were bilateral and/or palpable tender lumps were benign, and 2 (1.6%) with bloody discharge from the nipple were malignant.

**Table 1:** Clinicopathologic characteristics of 125 breast masses in males.

Variables	N (%)	Benign (%)	Malignant (%)	P value
**Mean of age ±SD**		56.68±20.51	73.16±12.63	< 0.001
**Laterality**	125			0.0037^a^
Unilateral	100(80)	75(75)	25(25)	
Bilateral	25(20)	25(100)	0(0)	
**Palpable lump**	125			1.0000^a^
Present	107(86)	85(79)	22(21)	
Absent	18(14)	15(83)	3(17)	
**Palpable tender lump**	125			0.0019^a^
Present	27(22)	27(100)	0(0)	
Absent	98(78)	73(74)	25(26)	
**Tender, nonpalpable**	125			0.6922^a^
Present	11(9)	10(91)	1(9)	
Absent	114(91)	90(79)	24(21)	
**Bloody discharge, nipple**	125			0.0387^a^
Present	2(2)	0(0)	2(100)	
Absent	123(98)	100(81)	23(19)	
**Serous discharge, nipple**	125			1.0000^a^
Present	2(2)	2(100)	0(0)	
Absent	123(98)	98(80)	25(20)	
**Swelling, nonpalpable**	125			0.5829^a^
Present	4(3)	4(100)	0(0)	
Absent	121(97)	96(79)	25(21)	

SD, standard deviation; N (%), number (percentage).

^a^ Fisher’s exact test.

### Ultrasound features

**Fig 2:**
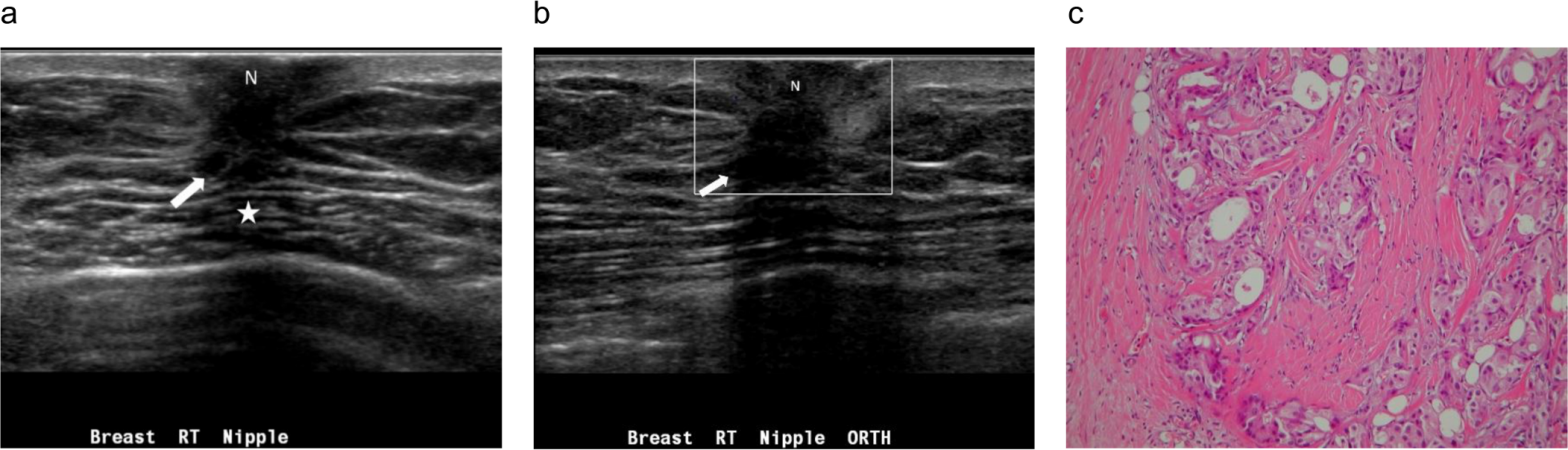
A palpable lump in the right breast of a 66-year-old man. (a) Radial ultrasound shows an irregular shape, a noncircumscribed margin, and a heterogeneously hypoechoic, subareolar, solid nodule (arrow). The nodule has a nonparallel orientation, an abrupt interface, posterior acoustic shadowing (star), and a concentric relationship to the nipple (N). There is no echogenic halo. (b) Color Doppler ultrasound shows an absence of vessels in the nodule, categorized as a low color flow signal (arrow). (c) Pathology specimen shows invasive ductal carcinoma with a surrounding fibrous reaction. Collagen fibers are arranged in laminated layers. There is no tumor necrosis. (Hematoxylin-eosin, original magnification ×200).

**Fig 3:**
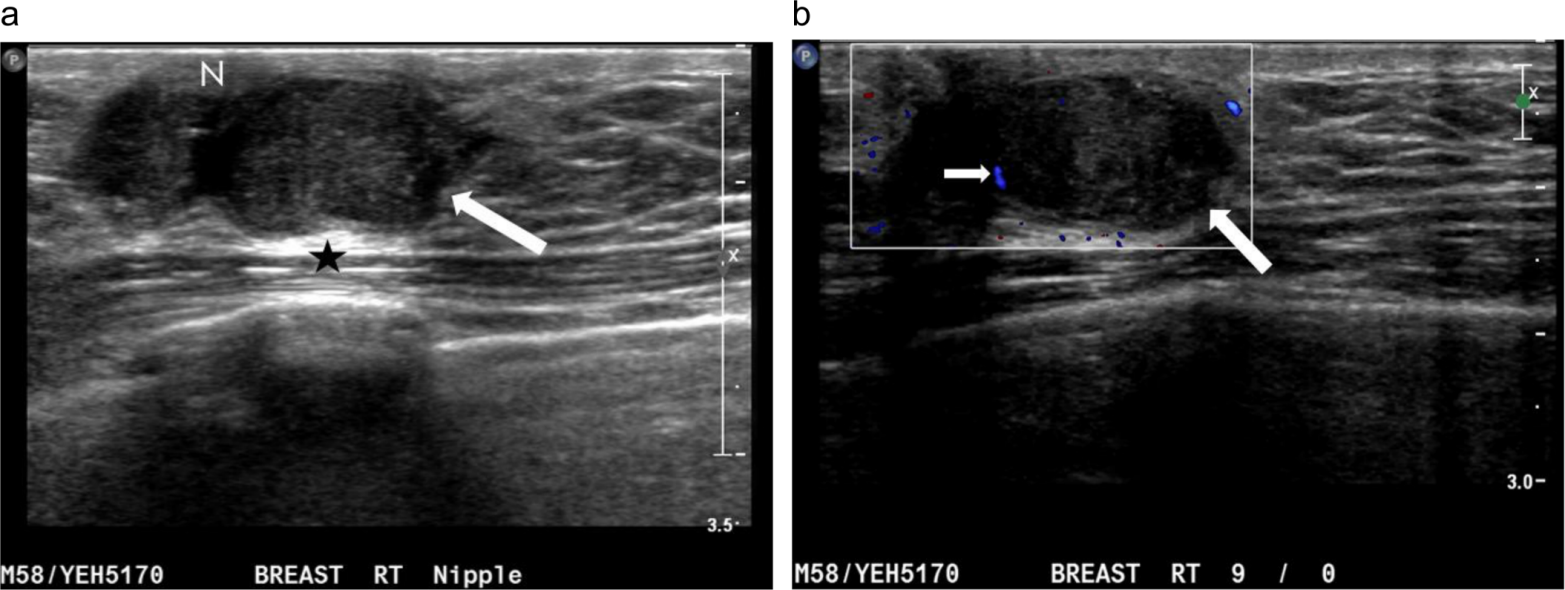
A palpable nodule in the right breast of a 58-year-old man. (a) Radial sonogram shows an irregular shape, a noncircumscribed margin, and a heterogeneously hypoechoic, subareolar nodule (arrow). The nodule has a parallel orientation and a mildly eccentric relationship to the nipple (N). There is posterior acoustic enhancement (black star). (b) Transverse color Doppler ultrasound shows a short color flow signal (short arrow) in the nodule (long arrow), categorized as low color flow with predominantly internal vascularity. The postoperative pathological diagnosis is invasive ductal carcinoma.

**Fig 4:**
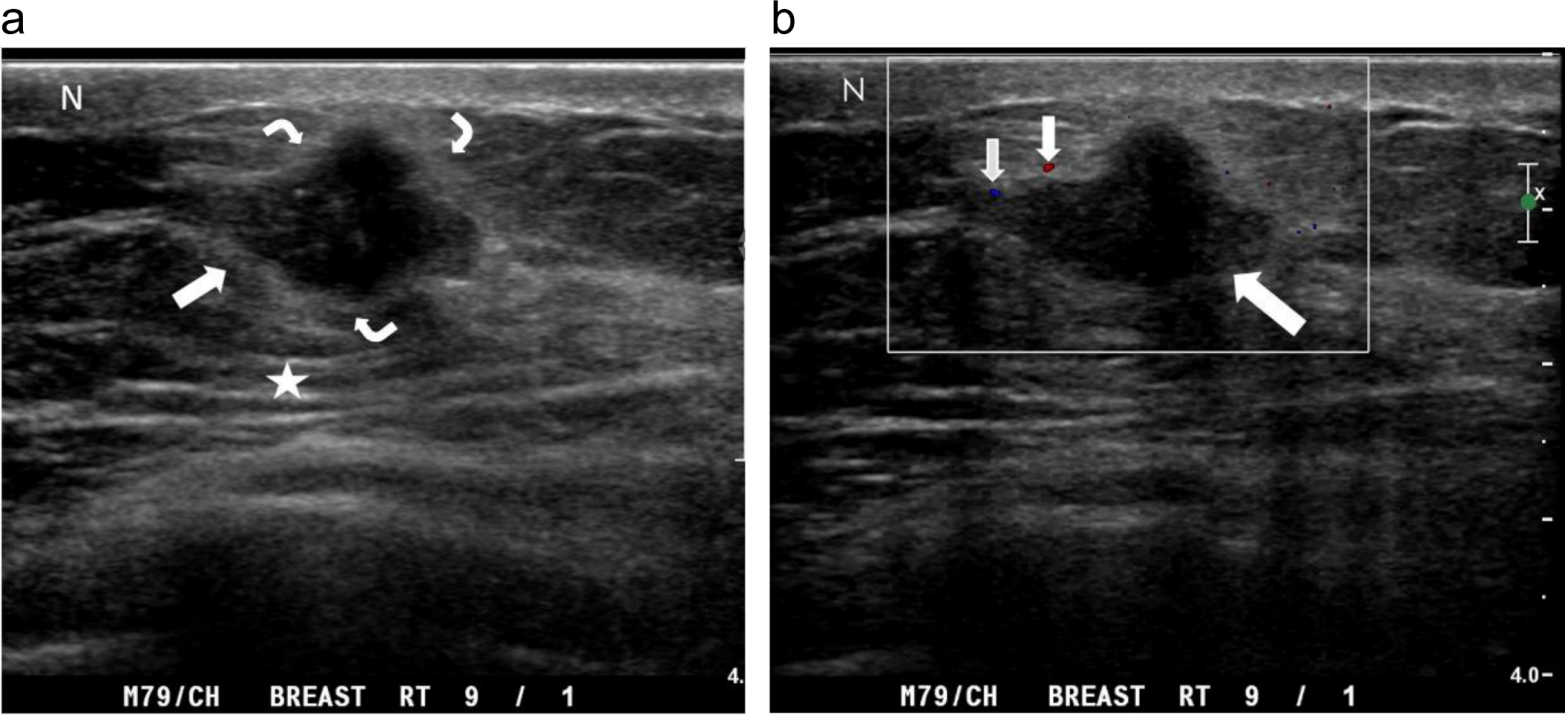
A palpable lump in the right breast of a 79-year-old man. (a) Radial ultrasound shows an irregular shape, a noncircumscribed margin, and a heterogeneously hypoechoic, subareolar, solid nodule (straight arrow). The nodule has a parallel orientation, an echogenic halo (curved arrows), posterior acoustic enhancement (star), and a markedly eccentric relationship to the nipple (N). (b) Color Doppler ultrasound shows transient occasional color pixels (short arrows) around the nodule (long arrow), classified as a low color flow signal with vessels predominantly in the rim. The postoperative pathological diagnosis is invasive ductal carcinoma.

**Fig 5:**
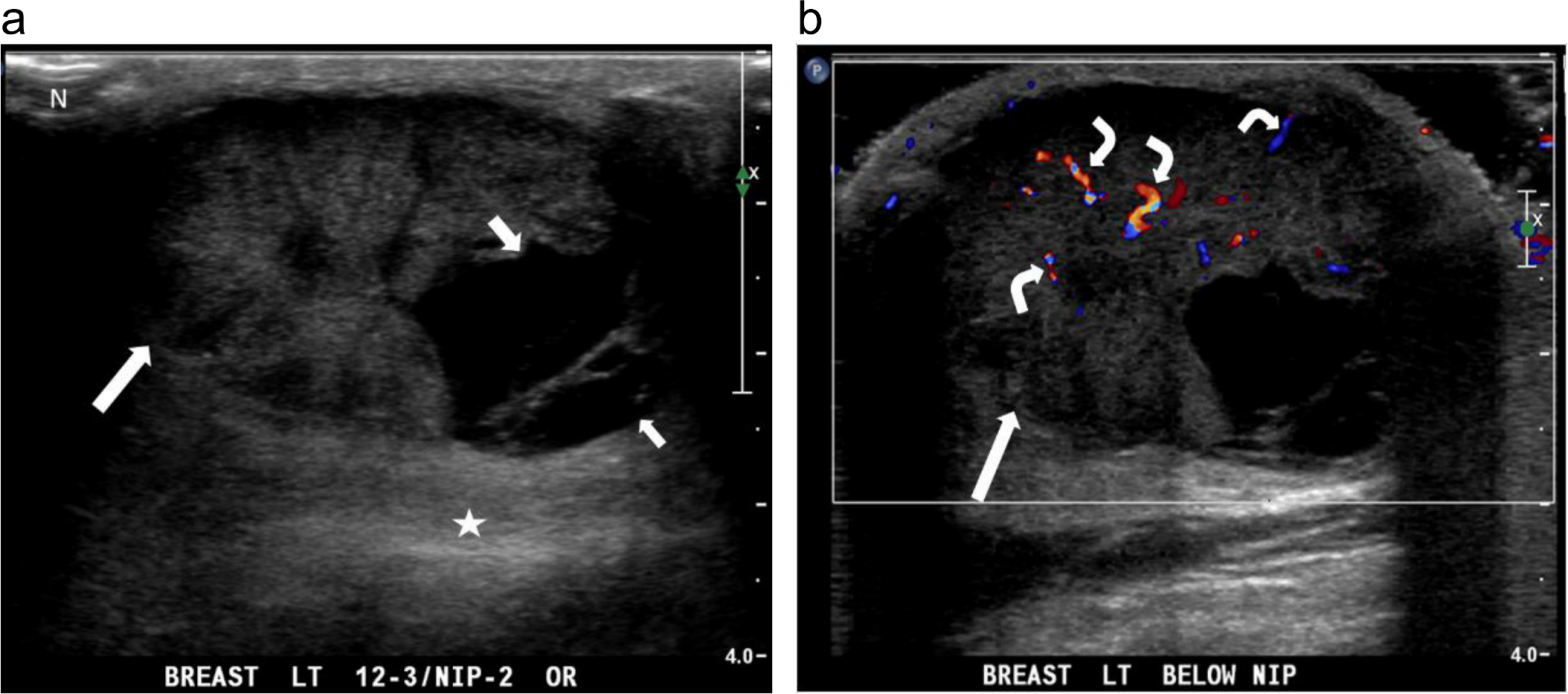
A palpable mass in the left breast of an 84-year-old man. (a) anti-radial ultrasound shows an oval-shaped, circumscribed mass (long arrow) with solid and cystic (small arrows) components. There is posterior acoustic enhancement (star) and a markedly eccentric relationship to the nipple (N). (b) Color Doppler ultrasound shows multiple color pixels and multiple pedicles of blood supply (curve arrows) in the mass, categorized as a rich color flow signal with predominantly internal vascularity. The postoperative pathological diagnosis is invasive ductal carcinoma.

**Fig 6:**
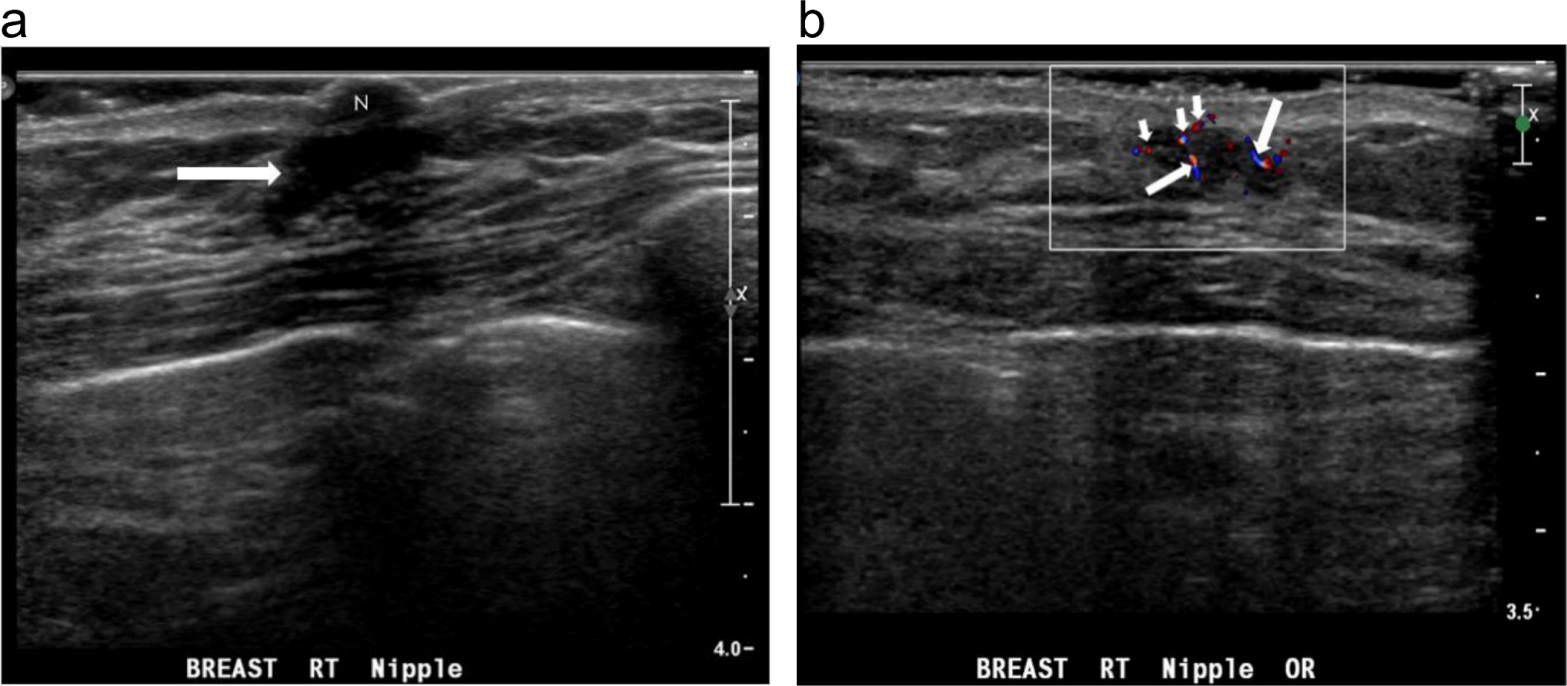
A nonpalpable nodule in the right breast of a 77-year-old man with bloody discharge from the right nipple. (a) Radial sonogram shows an irregular shape, a noncircumscribed margin, a mildly eccentric, homogeneously hypoechoic, subareolar nodule. The nodule has a parallel orientation and an abrupt interface. Neither an echogenic halo nor posterior features are present. (b) Transverse color Doppler ultrasound shows multiple color pixels (short arrows) and short pedicles of blood supply (long arrows) in the nodule, categorized as a rich color flow signal with predominantly internal vascularity. The postoperative pathological diagnosis is ductal carcinoma *in situ*.

**Fig 7:**
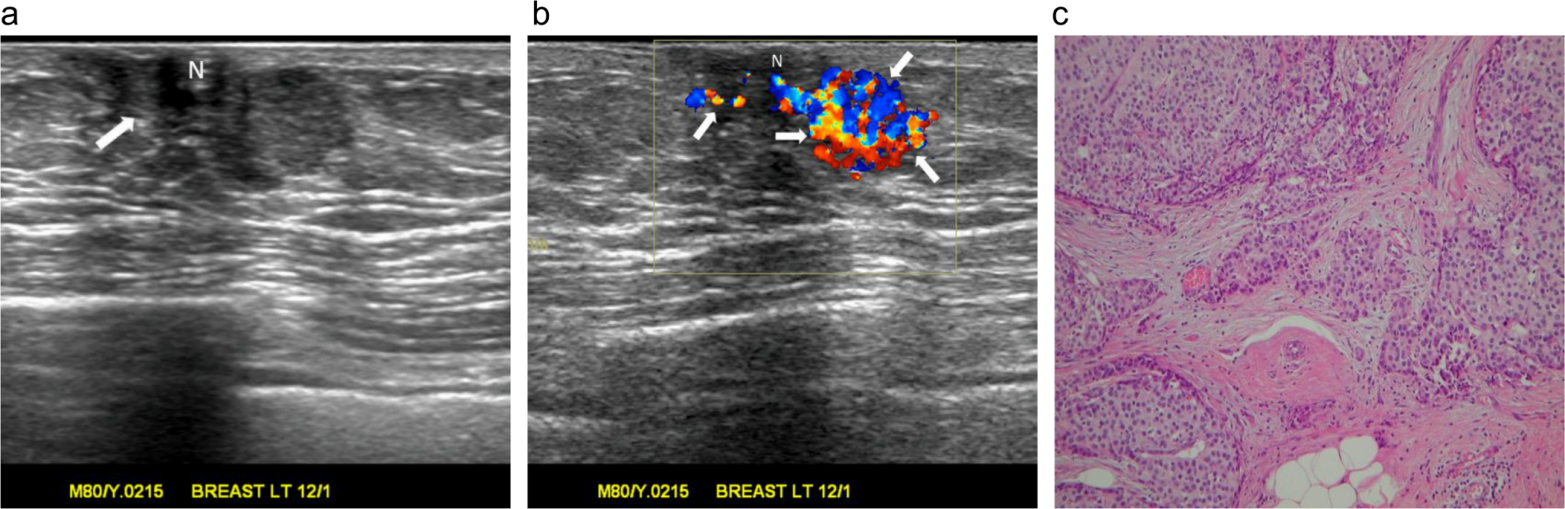
A palpable lump in the left breast of an 80-year-old man. (a) Radial sonogram shows an irregular shape, a noncircumscribed margin, a heterogeneously hypoechoic, subareolar, solid nodule (arrow). The nodule has a parallel orientation (arrow) and a concentric relationship to the nipple (N). There is an abrupt interface. (b) Color Doppler ultrasound shows multiple color pixels and pedicles of blood supply (arrows) pooling in the nodule, classified as a rich color flow signal with predominantly internal vascularity. (c) Pathology specimen shows an invasive papillary carcinoma with tissue invasion and fibrous reaction. There is no tumor necrosis. (Hematoxylin-eosin, original magnification ×100).

**Fig 8:**
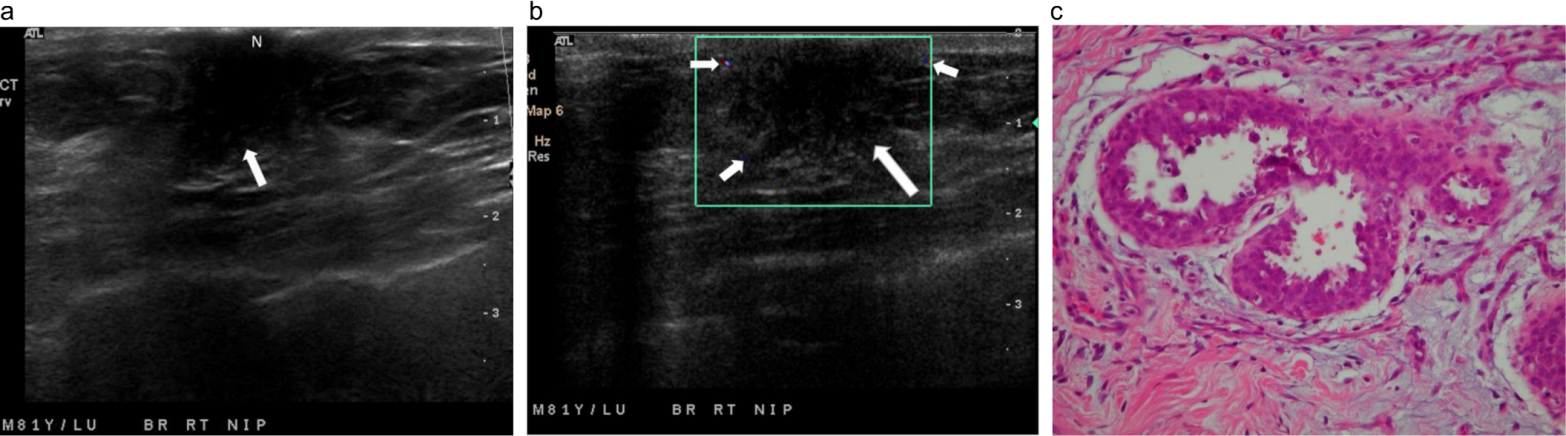
A palpable tender lump in the right breast of an 81-year-old man. (a) Radial ultrasound shows an irregular shape, a noncircumscribed margin, and a homogeneously hypoechoic, subareolar, solid nodule (arrow). The nodule has a nonparallel orientation and a concentric relationship to the nipple (N). (b) Color Doppler ultrasound shows occasional transient pixels (short arrows) around the nodule (long arrow), classified as a low color flow signal with flow predominantly in the rim. (c) Pathology specimen shows gynecomastia characterized by intraductal epithelial hyperplasia and proliferation of periductal collagenous connective tissue (hematoxylin-eosin, original magnification ×100).

**Fig 9:**
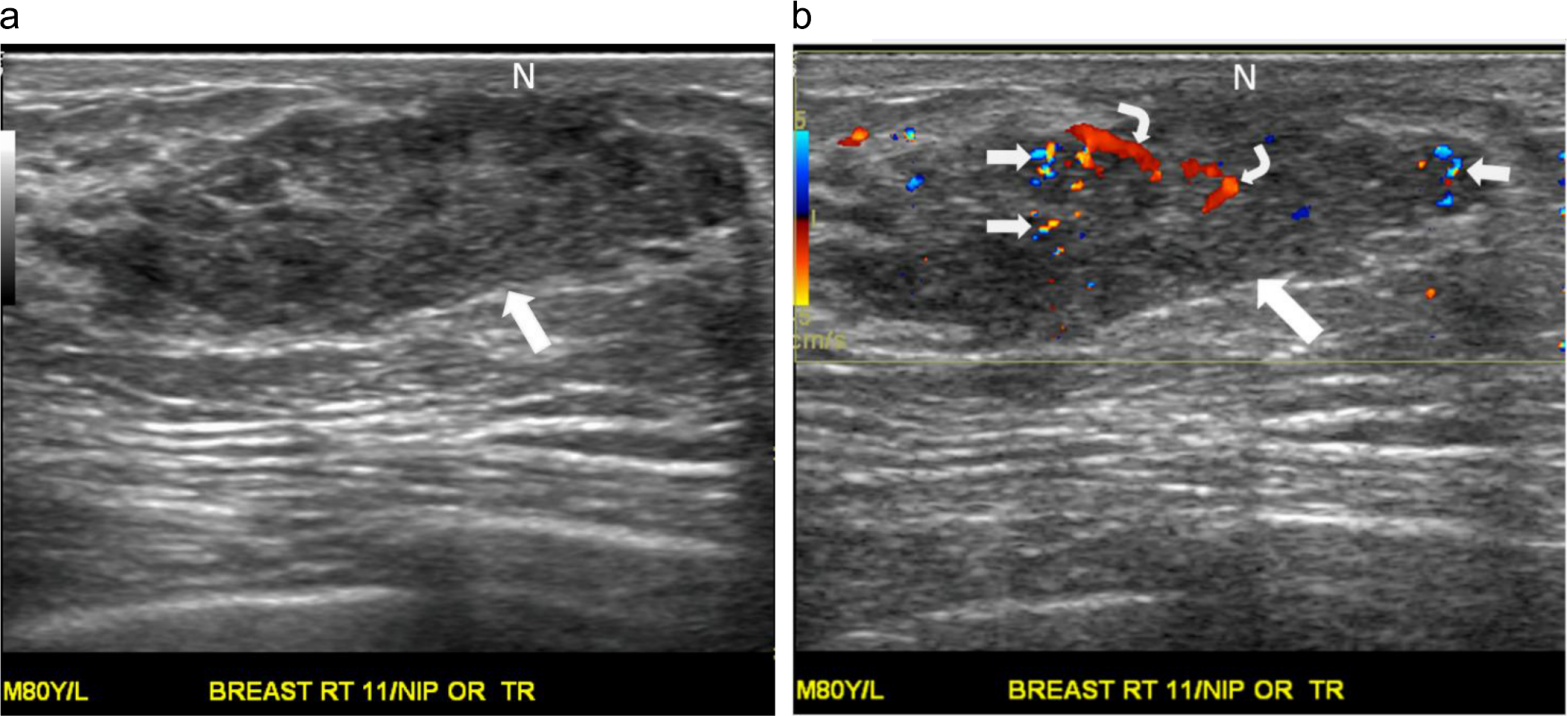
A palpable mass in the right breast of an 80-year-old man. (a) Transverse ultrasound shows an oval shape, a noncircumscribed margin, and a heterogeneously hypoechoic, solid nodule (arrow). The nodule has a parallel orientation and a mildly eccentric relationship to the nipple (N). (b) Color Doppler ultrasound shows multiple color pixels (short arrows) and two pedicles of blood supply (curve arrows) in the nodule (long arrow), categorized as a rich color flow signal with predominantly internal vascularity. The postoperative pathological diagnosis is gynecomastia.

**Fig 10:**
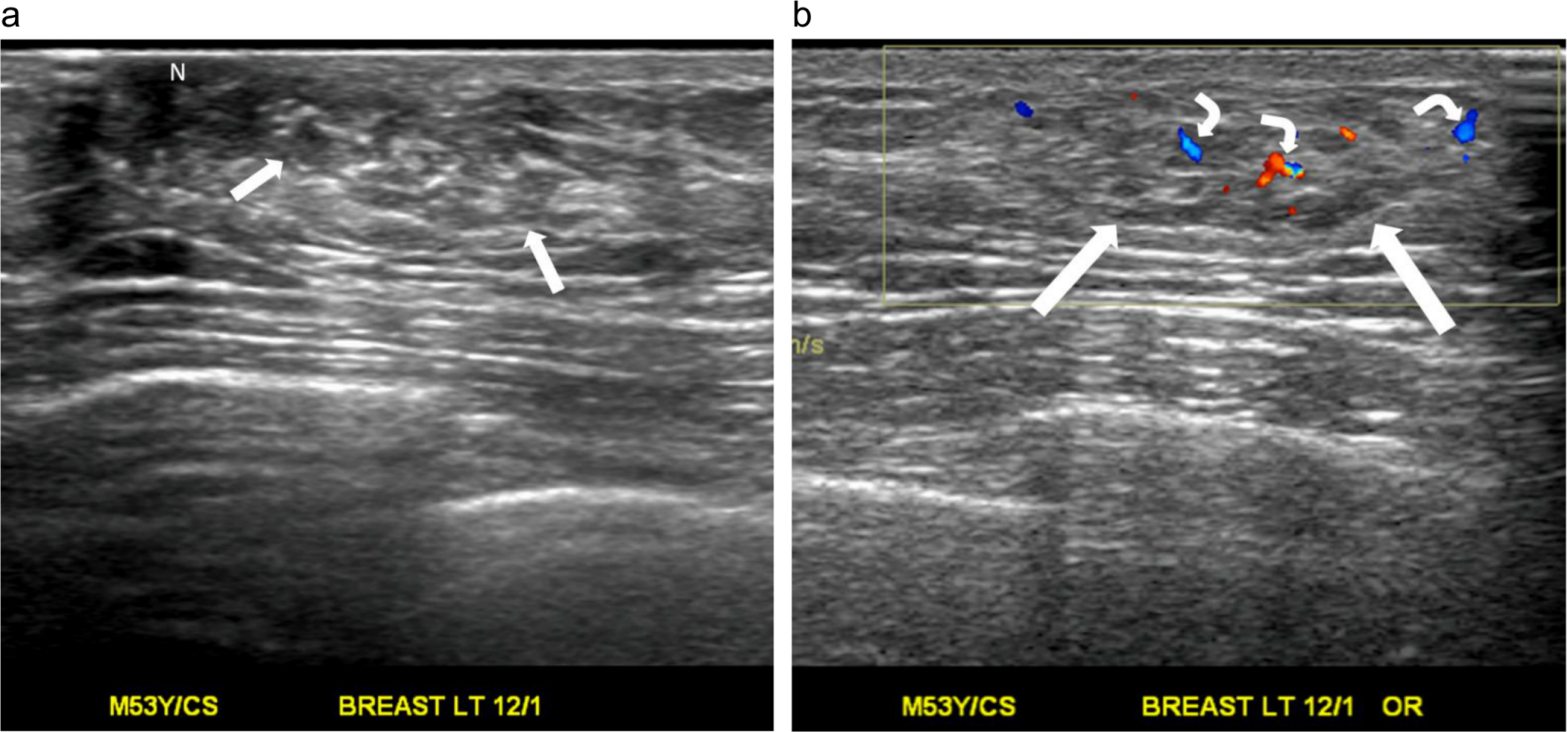
A palpable lump in the left breast of a 53-year-old man. (a) Radial ultrasound shows an irregular shape, a noncircumscribed margin, and a heterogeneously isoechoic, solid nodule (arrows). The nodule has a parallel orientation and a markedly eccentric relationship to the nipple (N). (b) Color Doppler ultrasound shows occasional transient color pixels and multiple short pedicles of blood supply (curve arrows) in the nodule (straight arrows), classified as a rich color flow signal with predominantly internal vascularity. The postoperative pathological diagnosis is gynecomastia.

**Fig 11:**
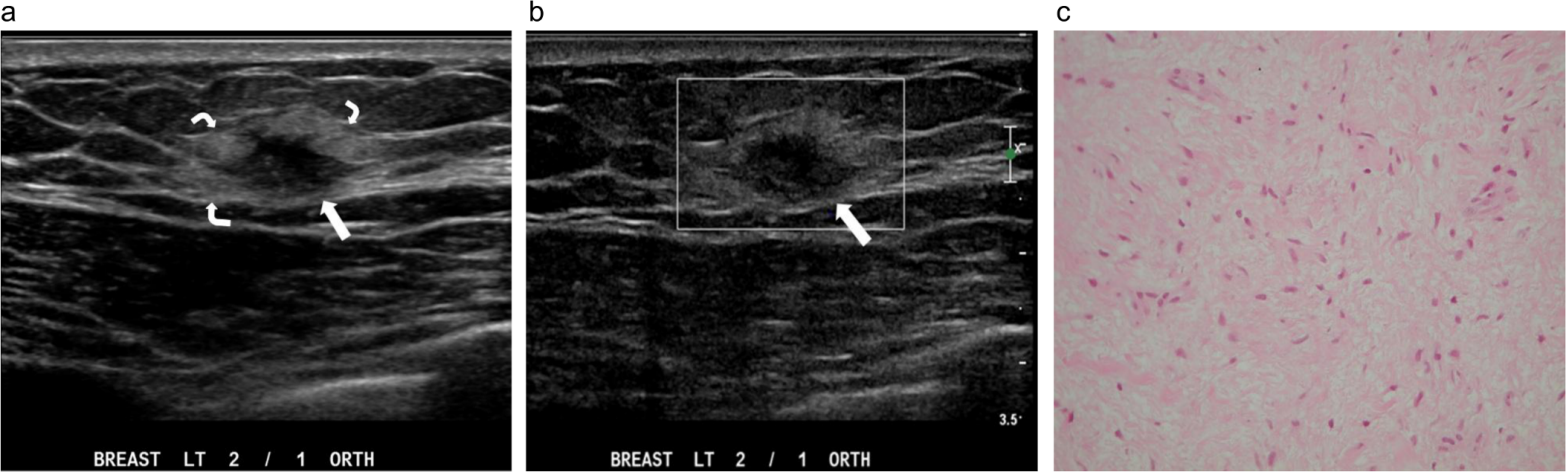
A palpable nodule in the left breast of a 55-year-old man. (a) Transverse ultrasound shows an irregular shape, a noncircumscribed margin, and a heterogeneously hypoechoic, solid nodule (straight arrow) with the center 1 cm away from the nipple. The nodule has an echogenic halo (curve arrows) and a markedly eccentric relationship to the nipple. (b) Color Doppler ultrasound shows absent color signal in the nodule (arrow), categorized as a low color flow signal. (c) Pathology specimen mainly shows fibroadipose stromal tissue with stromal fibrosis, myxoid degeneration, and focal fatty necrosis. No mammary gland tissue or malignant cells can be identified. (Hematoxylin-eosin, original magnification ×200).

Table 2 summarizes the gray-scale US findings of all 125 breast lesions. The features of the masses on color Doppler ultrasonography are summarized in Table 3. Mildly or markedly eccentric to nipple (P = 0.044), round or irregular shape (P = 0.044), presence of an echogenic halo (P < 0.001), predominantly internal vascularity (P = 0.006), and rich color flow signal (P < 0.001) were significantly associated with malignancy (Figs 2–7). At least 1 of 5 statistically significant US features was present in each malignancy. In addition, the mean maximum diameter, echotexture, echo pattern, heterogeneity, margins, orientation, and posterior acoustic features were not helpful for distinguishing malignant (Figs 2–7) from benign masses (Figs 8–11).

**Table 2:** The features of 125 breast masses in males on gray-scale ultrasonography.

Feature	N (%)	Benign (%)	Malignant (%)	P value
**MMD** ± **SD**, mm		19.97 ± 11.17	24.29 ± 13.84	0.103
**Relationship to nipple**	125			0.044
Concentric	56 (45)	50 (89)	6 (11)	
Mildly eccentric	24 (19)	16 (67)	8 (33)	
Markedly eccentric	45 (36)	34 (76)	11 (24)	
**Echotexture**	125			0.464^a^
Cystic	1 (1)	1(100)	0 (0)	
Solid and cystic (mixed)	5 (4)	3 (60)	2 (40)	
Solid	119(95)	96 (81)	23 (19)	
**Echo pattern**	125			0.710^a^
Anechoic	1 (1)	1 (100)	0 (0)	
Hypoechoic	110(88)	88 (80)	22 (20)	
Isoechoic	2 (2)	2 (100)	0 (0)	
Hyperechoic	7 (6)	6 (86)	1 (14)	
Complex	5 (4)	3 (60)	2 (40)	
**Heterogeneity**	125			0.088
Predominantly homogeneous	69 (55)	59 (86)	10 (14)	
Heterogeneous	56 (45)	41 (73)	15 (27)	
**Shape**	125			0.044^a^
Round	4 (3)	2 (50)	2 (50)	
Oval	43 (34)	39 (91)	4 (9)	
Irregular	78 (62)	59 (76)	19 (24)	
**Margin**	125			0.177
Circumscribed	39 (31)	34 (87)	5 (13)	
Non-circumscribed	86 (69)	66 (77)	20 (23)	
**Boundary**	125			<0.001^a^
Abrupt interface	108(86)	92 (85)	16 (15)	
Echogenic halo	17(14)	8 (47)	9 (53)	
**Orientation**	125			0.618
Parallel	90 (72)	73 (81)	17 (19)	
Nonparallel	35 (28)	27 (77)	8 (23)	
**Posterior acoustic feature**	125			0.632
Normal	85 (68)	70 (82)	15 (18)	
Enhancement	36 (29)	27 (75)	9 (25)	
Shadowing	4 (3)	3 (75)	1 (25)	

MMD, mean maximum diameter; SD, standard deviation; N (%), number (percentage);.

^a^Fisher’s exact test.

**Table 3:** The features of 125 breast masses in males on color Doppler ultrasonography.

Feature	N (%)	Benign (%)	Malignant (%)	P value
**Color Flow Distribution**	125			0.006
Absent	49 (39)	46 (94)	3 (6)	
Vessels predominantly in the rim	25 (20)	19 (76)	6 (24)	
Predominantly Internal vascularity	51 (41)	35 (69)	16 (31)	
**Color Flow Grading**	125			<0.001
Low color flow signal	93 (74)	82 (88)	11 (12)	
Rich color flow signal	32 (26)	18 (56)	14 (44)	

N (%), number (percentage).

As shown in Table 4, multivariate logistic regression analysis identified echogenic halo (OR = 3.955; 95% CI: 1.005–15.564; P = 0.049) and the presence of a rich color flow signal (OR = 8.330; 95% CI: 1.505–46.102; P = 0.015) as independent factors for malignancy. These 2 independent risk factors accounted for 19 (76%) of 25 malignancies.

**Table 4:** Multivariate logistic regression analysis of ultrasonographic features of male breast malignancies.

Features	OR (95% CI)	P value
**Relationship to nipple**		
Concentric	1	
Mildly eccentric	2.961 (0.707–12.396)	0.137
Markedly eccentric	3.065 (0.789–11.910)	0.106
**Shape**		
Oval	1	
Round	8.340 (0.590–117.927)	0.117
Irregular	2.564 (0.661–9.938)	0.173
**Lesion boundary**		
Abrupt interface	1	
Echogenic halo	3.955 (1.005–15.564)	0.049
**Color flow Distribution**		
Absent	1	
Vessels predominantly in the rim	1.999 (0.389–10.281)	0.407
Predominantly internal vascularity	1.159 (0.161–8.334)	0.884
**Color flow Grading**		
Low color flow signal	1	
Rich color flow signal	8.330 (1.505–46.102)	0.015

Table 5 summarizes the histopathological results of the 125 lesions. Twenty-five of the 125 masses (20%) were malignant (Figs 2–7) and 100 (80%) were benign (Figs 8–11). The most common malignancy was invasive ductal carcinoma (17/25, 68%) (Figs 2–5), whereas gynecomastia was the most common type of benign mass (53/100, 53%) (Figs 8–10).

**Table 5:** The histopathological diagnosis of 125 breast masses in males.

Histopathological diagnosis	N (%)
**Benign Mass**	
Gynecomastia	53 (42.4%)
Pseudogynecomastia	2 (1.6%)
Chronic inflammation	7 (5.6%)
Cystic Lymphangioma	1 (0.8%)
Vasculitis	1 (0.8%)
Nodular fasciitis	1 (0.8%)
Abscess	2 (1.6%)
Epidermal cyst	6 (4.8%)
Epithelial hyperplasia	3 (2.4%)
Lipoma	4 (3.2%)
Angiolipoma	2 (1.6%)
Neurofibroma	2 (1.6%)
Reactive hyperplasia of a lymph node	1 (0.8%)
Myofibroblastoma	2 (1.6%)
Stromal fibrosis	4 (3.2%)
Fibroadipose tissue	5 (4.0%)
Normal breast tissue	4 (3.2%)
**Malignant Mass**	
Invasive ductal carcinoma	17 (13.6%)
Ductal carcinoma in situ (DCIS)	1 (0.8%)
Intraductal papilloma with DCIS	2 (1.6%)
Invasive papillary carcinoma	1 (0.8%)
Apocrine adenocarcinoma	1 (0.8%)
Metastatic adenocarcinoma	1 (0.8%)
Neuroendocrine carcinoma	1 (0.8%)
Metastatic carcinoma	1 (0.8%)
**Total**	125 (100%)

N (%), number (percentage)

Of the 12 patients with synchronous bilateral benign masses, 1 had bilateral pseudogynecomastia in the subareolar area, 1 had epidermal cysts in each breast, and the other 10 had bilateral gynecomastia, of whom 1 also had a benign palpable lymph node in the left breast near the axilla.

As shown in Table 1, being palpable was not different for benign versus malignant masses (79% vs 21%, P = 1.0, Fisher’s exact test). The 3 nonpalpable malignant masses included: 1 ductal carcinoma *in situ* with bloody nipple discharge, 1 intraductal papilloma with ductal carcinoma *in situ* presenting with bloody nipple discharge, and 1 invasive ductal carcinoma presenting with left breast with tenderness. Furthermore, 1 palpable tender mass with serous nipple discharge was chronically inflamed tissue. A nonpalpable lipoma presented with serous discharge from the right nipple, while the other nonpalpable benign masses presented with focal breast swelling and/or tenderness on physical examination.

As shown in Table 2, relative to the position of the nipple, of the 50 benign lesions with concentric features 38 (76%) were gynecomastia (Fig 8) and 12 (24%) were not. Of 16 benign conditions with mildly eccentric features, 10 (63%) were gynecomastia (Fig 9) and 6 (38%) were not. Of 34 conditions with markedly eccentric features, 5 (15%) were gynecomastia (Fig 10) and 29 (85%) were not (Fig 11). One cystic mass was a benign lymphangioma with an anechoic pattern. Of the 5 lesions with solid and cystic echotexture, 3 (60%) were benign masses including 2 abscesses and 1 area of chronic inflammation, and 2 (40%) were malignant and both were invasive ductal carcinomas (Fig 5). Five masses with a complex echo pattern were non-gynecomastia lesions with solid and cystic texture.

Of the 39 oval lesions that were benign, 22 (56%) were gynecomastia (Fig 9) and 17 (44%) were not. Of 59 masses with an irregular shape, 31 (53%) were gynecomastia (Figs 8 and 10), and 28 (47%) were benign non-gynecomastia (Fig 11). Of 4 round masses, the 2 (50%) benign round masses were a myofibroblastoma and an abscess. The 8 benign conditions with an echogenic halo included 2 cases of gynecomastia, 1 of reactive hyperplasia of a lymph node, 1 epidermal cyst with rupture, 1 neurofibroma, 1 myofibroblastoma, 1 case of nodular fasciitis, and 1 of stromal fibrosis (Fig 11). Of 27 benign lesions with nonparallel orientation, 7 (26%) lesions were gynecomastia and 20 (74%) were not. In terms of posterior acoustic features, of 27 benign lesions with posterior acoustic enhancement, 9 (33%) masses were gynecomastia and 18 (67%) were not. Of 3 benign lesions with shadowing, all (100%) were gynecomastia.

As shown in Table 3, among 46 benign masses with absent color flow vascularity, 26 (57%) were gynecomastia and 20 (44%) were not (Fig 11). Of 19 with vessels predominantly in the rim, 6 (32%) were gynecomastia (Fig 8) and 13 (68%) were not. Of 35 with predominantly internal vascularity, 21 (60%) were gynecomastia (Figs 9 and 10) and 14 (40%) were not. Of 82 benign lesions with low color flow signal, 44 (54%) were gynecomastia and 38 (46%) were not. Moreover, 9 (50%) of 18 masses with rich color flow signal were gynecomastia.

## Discussion

Male breast cancer is much less common than gynecomastia and other benign breast pathologies [4], which makes it challenging for clinicians to differentiate malignancies from benign conditions in men with breast masses. Benign conditions of the male breast usually need only conservative therapy, but breast malignancies require timely surgical intervention. Clinical examination is the first step towards evaluating male breast masses. In our study, advanced age (mean age 73.16 vs. 56.68 years, P < 0.001) and bloody discharge from the nipple (P = 0.0387) were significant clinical features associated with malignancy. Synchronous bilateral lesions and palpable tender lumps were more likely to be benign than malignant. Of the 125 mases, 50 (40%) that were bilateral and/or palpable tender lumps were benign; 2 (1.6%) with bloody discharge from nipple were malignant. Among US features, being eccentric to a nipple, having an irregular shape, the presence of an echogenic halo, the presence of internal vascularity, and a rich color flow signal were all significantly associated with malignancy. Moreover, the presence of an echogenic halo and a rich color flow signal were independent predictors of breast malignancy. Six (4.8%) of 125 masses with anechoic cystic echotexture or complex solid and cyst features were all non-gynecomastia lesions.

Clinical presentations in concert with US assessment may help the evaluation and management of male breast masses. Gynecomastia or non-gynecomastia benign masses usually present as palpable tender lesions [1, 4]. Synchronous, bilateral nodules in the subareolar areas are present in approximately half of patients with gynecomastia [7]. Our research results are compatible to those of prior studies that suggested US might not be necessary for bilateral and/or palpable tender lumps (50/125, 40%) in male breasts (P < 0.05). Correcting underlying cause of gynecomastia and close follow-up might be a good strategy for managing bilateral and/or palpable tender lumps [4]. In converse, male breast cancer usually presents as an unilateral, palpable, and painless lump [1]. The incidence of synchronous bilateral breast cancers is in men reported to be only 1.5% to 2% of all male breast cancers [13]. Moreover, bloody nipple discharge or retraction and axillary lymphadenopathy might be present in the case of malignancy [1]. Bloody nipple discharge is found in 25% of men with breast cancers [5]. In our study, 2 of 2 masses presenting as bloody nipple discharge were malignant (P = 0.0387). Previous reports have shown that the mean age at diagnosis of male breast cancer is 67–69 years [6, 14, 15, 16], which is younger than the mean age of our patients (73.16 years). Timely biopsy might be performed when the male breast masses (75/125, 60%) exhibit significantly malignant clinical and US features, such as advanced age, bloody discharge from the nipple, presence of an echogenic halo, and a rich blood flow signal. However, when conservative treatment for any mass is not effective, biopsy or excision should be performed [4].

The relationship between US features of malignant breast lesions and pathological characteristics of breast cancers have been reported [5, 6, 8, 17–19]. An irregular shape and noncircumscribed margins on US suggest tumor invasion into surrounding tissue. Nonparallel orientation may represent tumor spread through tissue-plane boundaries [20]. Posterior acoustic shadowing may indicate a desmoplastic reaction in breast cancers. More highly vascular lesions and intralesional color flow signals on CDU are compatible with increased angiogenesis in breast malignancies [18].

Gynecomastia in its early stage is characterized histologically by proliferation of intraductal epithelium, periductal inflammation, and surrounding edema, which clinically corresponds to a painful breast mass and usually appears on US as a hypoechoic oval nodule with a well circumscribed margin in the subareolar region [14]. However, late stage gynecomastia has dilated ducts surrounding stromal fibrosis, and usually presents as a hypoechoic lesion with an irregular, ill-defined, or macrolobulated margin [5, 14]. In our study, some masses in cases of gynecomastia (Figs 8–10) had various projections, which could develop a nonparallel orientation, and noncircumscribed margins mimicking breast cancer. These particular US features, therefore, were not helpful in our series for differentiating malignant from benign conditions. The situation is different in women, where the 3 most common breast masses are simple cysts, fibrocystic changes, and fibroadenoma [21, 22]. These usually present with typical benign US features with high predictive value: an oval shape, circumscribed margins, an abrupt interface, and a homogeneous echo texture.

Previous studies have demonstrated that heterogeneity and hypoechoic features are not useful in differentiating benign from malignant male breast lesions [2, 6, 23]. Hyperechogenicity is thought to be a reliable predictor of benignity, but there was a malignant mass in our series with heterogeneous hyperechogenicity. Posterior acoustic enhancement has been reported to be associated with relative preservation of the transmitted ultrasound beam distal to a mass [24]. Fluid, mucin, hemorrhage, or necrosis usually results in the appearance of posterior acoustic enhancement [25, 26]. Conversely, Maturen et al. considered that fluid within a mass was not required for posterior acoustic enhancement [24]. Any mass with a simple internal architecture might have posterior enhancement on US, which may be why in our study the appearance of posterior features did not differ significantly between malignant and benign lesions.

In terms of the relationship of male breast masses to the nipple, we found that lesions mildly or markedly eccentric from nipple were significantly associated with cancer; in contrast, masses concentric to the nipple were significantly more likely to be benign (P = 0.044). Prior studies have also suggested that gynecomastia usually appears as a concentric subareolar mass, while other benign masses and cancers in men are usually eccentric to the nipple-areolar complex [14, 27]. However, eccentricity to the nipple was present in some cases of gynecomastia in our study, as well as with a number of other benign lesions, so the relationship of the lesion to the nipple was not a statistically significant independent predictor of malignancy.

Chen at el. recommended that a mass in the male breast with a cystic component on US should be considered suspicious for malignancy [6]. Chau at al. reported that invasive or *in situ* papillary cancers in the male breast often had complex mixed cystic and solid composition on US [15]. In our study, 2 (40%) of 5 male breast lesions with cystic and solid components were invasive ductal carcinomas. The only invasive papillary carcinoma in our series did not have mixed components. A small number of benign conditions (3/100, 3%), 1 case of chronic inflammation and 2 abscesses also had cystic and solid US features. Our findings thus suggest that mixed components on US are not a feature specific for male breast cancers, and are not present in gynecomastia.

The histopathological findings associated with an echogenic halo indicate the invasion of malignant cells into fat tissue, where they exist along with adipocytes and elastic fibers [19]. Abscesses can also display an echogenic halo, which may be related to an inflammatory reaction and edema seen on histopathological examination. An echogenic halo is considered to be of no diagnostic significance for breast malignancy in the 2013 BI-RADS Atlas (5^th^ edition). However, our findings suggest that an echogenic halo was not only a significant feature, but also an independent US predictor for male breast malignancy. In our study, 8 (47%) of 17 masses were benign lesions, including 2 cases of gynecomastia, and had an echogenic halo. Conversely, there was no obvious echogenic halo around 2 abscesses in the current series.

Some studies [28, 29] have found that vascular characteristics were the best diagnostic determinant in women to distinguish malignant from benign breast lesions. Benign breast lesions either had no vascularization, or had vessels around the margin of the lesions, while breast cancers had multiple vessels penetrating into, or blood pooling in the lesions [28–30]. However, our study showed that absent vascularization, predominantly in the rim or predominantly internal vascularity, could be present in benign male breast masses. The feature of predominantly internal vascularity was significantly more common in malignancies than in benign masses, but on multivariate analysis it was still not an independent predictor of cancer. On the other hand, a rich color flow signal was an independent predictor of malignancy (OR = 8.330, CI: 1.505–46.102, P < 0.001).

This retrospective study has some bias. Ultrasound is a dynamic study, and very much operative dependent. Information bias is introduced in our study when using the static US images saved by technicians that performed the examination. Selection bias occurred when the question selecting patients with the preoperative ultrasonic examination and the pathological results thus missing many examinations. However, it is very difficult to accumulate enough cases of a rare condition like male breast cancer.

This study also has several limitations. First, it is a retrospective study with a relatively small number of patients. Only biopsy-proven diagnoses of masses in patients who had had preoperative US were included. Had cases without US had been included, the findings might have differed somewhat. Second, because of the retrospective nature data recorded in the charts may have been incomplete. For this reason, we did not evaluate other risk factors for male breast cancer including prior radiation therapy of the chest, smoking and alcohol history, liver disease, hyperestrogenism, androgen deficiency, undescended testes, orchitis, congenital inguinal herniation, Klinefelter’s syndrome, or family history of breast cancer [6, 27, 31]. Third, evaluation of microcalcifications and axillary lymph nodes were not included in this study. Fourth, we did not compare US to mammography. Last, because of incomplete data a detailed correlation between the US findings and clinical presentation of benign and malignant lesions, including onset, duration, skin changes, and other associated symptoms and signs could not be investigated.

## Conclusions

In conclusion, clinicians might use an integration of clinical and US findings to evaluate and manage a male breast lesion. Advanced age, bloody discharge from the nipple, a mass eccentric to a nipple, irregular shape, the presence of an echogenic halo, predominantly internal vascularity, and rich color flow signal on CDU were features more common in cancers than in benign lesions. The presence of an echogenic halo and rich color flow signal were independent US predictors of male breast cancers on multivariate analysis. The abovementioned specific clinical and US features of male breast cancers suggest that a biopsy or surgical excision be performed. Conversely, synchronous bilateral subareolar and/or palpable tender masses were almost always benign, and conservative follow-up may be appropriate. However, when conservative management is not effective, surgery should be considered.
